# Creating nanocrystallized chemotherapy: the differences in pressurized aerosol chemotherapy (PAC) via intracavitary (IAG) and extracavitary aerosol generation (EAG) regarding particle generation, morphology and structure

**DOI:** 10.7150/jca.39097

**Published:** 2020-01-01

**Authors:** Tanja Khosrawipour, Justyna Schubert, Joanna Kulas, Pawel Migdal, Mohamed Arafkas, Jacek Bania, Veria Khosrawipour

**Affiliations:** 1 Division of Colorectal Surgery, Department of Surgery, University of California, Irvine, California, USA.; 2 Department of Surgery (A), University-Hospital Düsseldorf, Düsseldorf, Germany; 3 Department of Food Hygiene and Consumer Health Protection, Wroclaw University of Environmental and Life Sciences, Wroclaw, Poland.; 4 Department of Biochemistry and Molecular Biology, Faculty of Veterinary Sciences, Wroclaw University of Environmental and Life Sciences, Wroclaw, Poland.; 5 Department of Environment, Hygiene and Animal Welfare, University of Environmental and Life Sciences, Wroclaw, Poland.; 6 Department of Plastic Surgery, Ortho-Klinik Dortmund, Dortmund, Germany.

**Keywords:** electron microscopy, pressurized intra-peritoneal aerosol chemotherapy (PIPAC), peritoneal metastases, nanoparticles, chemotherapy

## Abstract

**Background**: Nanocrystallization is a promising field for the development of new drugs. This study aims to present the use of nanocrystallization via intraperitoneal nanoaerosol therapy (INAT) for the treatment of peritoneal metastases.

**Methods**: A continuous aerosol generation device was used to aerosolize a highly concentrated doxorubicin solution within a dry CO_2_ environment. The produced nanoaerosol was directed into an *ex vivo* abdominal model and collision of aerosol particles with placed samples was subject to further analysis via scanning-electron microscopy (SEM). SEM detected structural changes of particles caused by migration to different locations.

**Results**: It was possible to visualize the contact of doxorubicin aerosol particles with the surface. Larger particles as well as particles closer to the aerosol generation chamber collided with the glass sample creating liquid drops, while smaller particles with more distance to the aerosol chamber collided as highly concentrated nanocrystals. The amount of nanocrystal particles outweighed the amount of fluid aerosol particles by far.

**Conclusions**: Under optimal conditions, the formation of nanocrystals via aerosol creation device is possible. While a wide range of possible applications of nanocrystals is conceivable, surface coating with drug particles is especially interesting as it may serve as an alternative to conventional liquid intraperitoneal chemotherapy. Further studies are required to investigate nanocrystallization of chemotherapeutic solutions as well as its physical and pharmacological properties and side effects.

## Introduction

Currently, nanocrystal formation is of major interest and often featured in technical and scientific studies regarding their possible future use in magnetic and electronic applications 1. The unique characteristics displayed by nanocrystals have also made them interesting for drug applications. Previous studies have demonstrated advantages of nanocrystals including high drug loading, enhanced dissolution and high bioavailability 2. Other studies focusing on pharmacologic properties, safety and drug delivery have predicted their great potential and clinical applicability 3-5. However, until today, the clinical application of nanocrystals in medicine remains uncommon.

Recent studies have introduced preliminary results of aerosol particle applications for oncologic treatment of peritoneal 6 and pleural metastases 7 as well as bladder cancer 8. The introduction of pressurized intraperitoneal aerosol chemotherapy (PIPAC) has also advanced efforts in investigating clinical applicability of nanocrystals. Within the last years, PIPAC has been increasingly implemented as a new treatment option for peritoneal metastases (PM). During PIPAC, a highly concentrated liquid chemosolution is applied into the abdominal cavity by an injection pump, which then transforms the liquid into an aerosol 9,10. The created aerosol droplets cover the peritoneal surface and create higher tissue penetration rates than achievable with conventional liquid solutions.

This phenomenon has been recently attributed to a “hyperconcentrated drug nanolayer” formed by aerosols which is believed to increase diffusion effects by creating a high concentration gradient on the drug surface and its underlying tissues 11. Interestingly, this effect can be achieved while total drug dosage is reduced compared to liquid intraperitoneal chemotherapy (IPC). Current research on chemoaerosol applications has advanced many new concepts investigating the potential of aerosol and drug nanoparticles and their unique characteristics. While PIPAC displays limitations 12, 13, its efficacy and potential benefits have also been recognized 14.

One of the first attempts to improve conventional PIPAC therapy was the recent introduction of hyperthermic intraperitoneal nanoaerosol therapy via extracavitary aerosol generator (EAG) by Göhler et al. 15. This study demonstrated that, by using intraperitoneal nanoaerosol therapy (INAT), far higher drug penetration rates were achievable compared to conventional PIPAC via micropump 9, 16 or microcatheter 17. However, the exact reasons for this improvement remain unknown.

EAG produces a smaller particle outburst than PIPAC via intracavitary aerosol generator (IAG) 15. These smaller particles supposedly create a smaller drug coating and hence result in lower in-tissue drug penetration rates, which have already been shown for PIPAC with IAG 11. However, the opposite is the case. Smaller particle sizes display a different relation between volume and particle surface. The special characteristics and physical properties of these smaller particles have not been recognized and the actual pharmacologic effects of these applied drugs have not been analyzed at all.

Since fluid nanoaerosol particles floating within the atmosphere are subdue too many outside influences, knowing their characteristics as well as their pharmacologic and physical properties is of high importance 18, 19. To investigate these micro- and nanoparticles in their function as doxorubicin-laden chemoaerosols, we conducted a structural particle analysis during surface collision and at different stages following aerosol formation.

**Figure 1:**
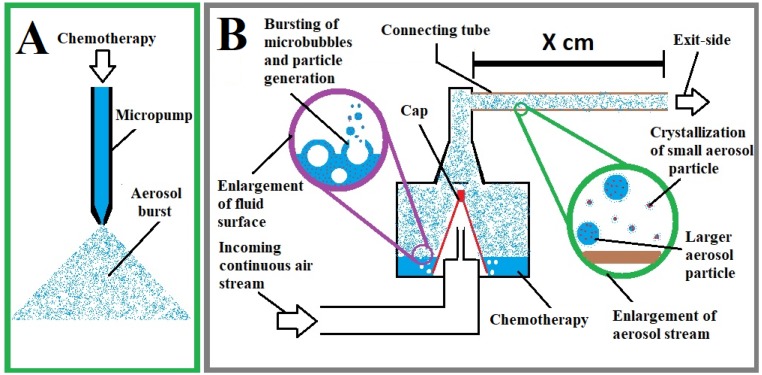
(A) PIPAC: model with an intracavitary aerosol generator (IAG). A continuous chemofluid is pressurized and directed through a microinjection pump which is placed in the abdominal cavity. (B) INAT: model with an extracavitary aerosol generator (EAG). A continuous air stream creates microbubbles in a liquid chemo filled cavity. As these microbubbles burst on the fluid surface, small chemo-laden aerosol particles are created and further transported with the airstream. During this airstream travel, aerosols crystallize. (C) X= 5cm, 20cm or 40 cm.

Furthermore, this study aims to discuss potential structural changes and explore if these represent a new form of drug particles. This study starts with an analysis of particles following outburst created by IAG device used in conventional PIPAC (Figure 1A). Then, analysis of particles following outburst from the EAG device is performed (Figure 1B).

## Material & Methods

### PIPAC model

The *ex vivo* PIPAC model has been well established and previously described in many studies 12, 13. PIPAC was performed with an intraperitoneal aerosol generator (IAG, Micropump®* Reger Medizintechnik, Rottweil, Germany*) and INAT was performed using an extraperitoneal aerosol generator (EAG, Medisana IN 500®, Medisana Neuss, Germany). A commercially available plastic box with a total volume of 3.5 liter, mimicking the abdominal cavity, was used. In the center of the plastic box cover, a 10 mm and a 5 mm trocar (Kii®Balloon Blunt Tip System, Applied Medical, Rancho Santa Margarita, CA, USA) were inserted. A round 5 mm diametric glass probe was placed at the rear of the box outside the main sprayjet on a plastic carrier. The plastic box was then tightly sealed and the CO_2_ capnoperitoneum was established and maintained for the entire PIPAC procedure. PIPAC was performed with 20 ml of a doxorubicin solution (1.2 mg/20 ml NaCl 0.9%) in a 12 mmHg capnoperitoneum.

### INAT model

#### Basic principle

The INAT procedure is conducted using the extraperitoneal aerosol generator (EAG). The basic concept of EAG is to create a “therapeutic” nanoaerosol extra-abdominally and then direct it into the abdominal cavity. Aerosol creation occurs via continuous airflow which is required for particle transport past the aerosol device and connecting tubes into the abdominal cavity.

#### Detailed description

A continuous air stream is directed into a chamber filled with liquid chemosolution, creating microbubbles within the liquid. Once these microbubbles reach the liquid surface, they burst and create micro- and nanodroplets which rise and float in the continuous air stream and ultimately leave via the side exit of the chamber (Figure 1).

For the INAT experiment the same commercially available hermetic plastic box was used. The nanoaerosol was directed into the box through a connecting plastic-tube and ultimately arrived in a plastic carrier where a round (Ø 5 mm) glass plate was placed. The probes were placed at different distances to the EAG by adding length to the connection tube. These probes were placed at 5cm, 20cm and 40cm distance to the EAG. 3ml (2mg/ml) of liquid doxorubicin is filled into a liquid reservoir of the EAG. The fluid doxorubicin solution is then aerosolized with a continuous gas stream of 5 liter/minute.

### Particle detection on scanning electron microscopy

All glass-probes were removed from the box. The surface of the glass-probes was analyzed via scanning electron microscopy (SEM). Samples were spotted on aluminum tables and dried, dusted with carbon (15 nm) and placed in the scanning chamber electron microscope (Auriga 60, Zeiss). All samples were carried out at a beam voltage equal to 2 kV and working distance 5 In Lens and SE2 secondary electron detectors.

### Statistical analyses

Experiments were independently performed in triplicate. Prism 7.0 software (GraphPad, La Jolla, CA, USA) was utilized to analyze the data. One-way ANOVA with multiple comparison test was used for analyses of independent groups. A significant p-value was considered at p<0.05.

## Results

**Figure 2:**
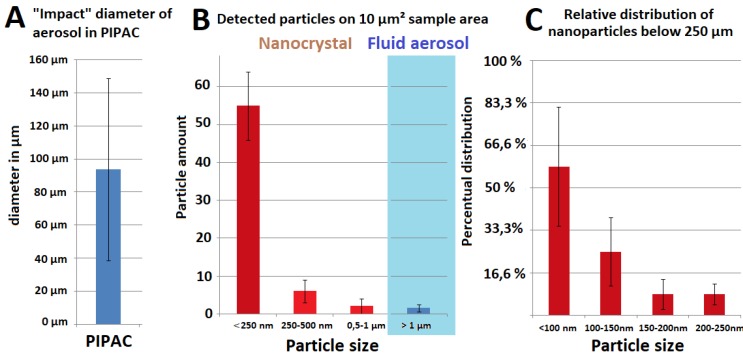
** (A)** Diameters of particles covering the glass probe after PIPAC with micropump in a 10mm2 sample area. **(B)** Total particle amount and size distribution on the glass probe following INAT at 40cm distance from the extracavitary aerosol generator (=” connecting tube”). Crystallized particles are illustrated in the red column. Fluid particles are illustrated in the blue column. **(C)** Percentual size distribution of main particle group (0 - 250nm).

**Figure 3:**
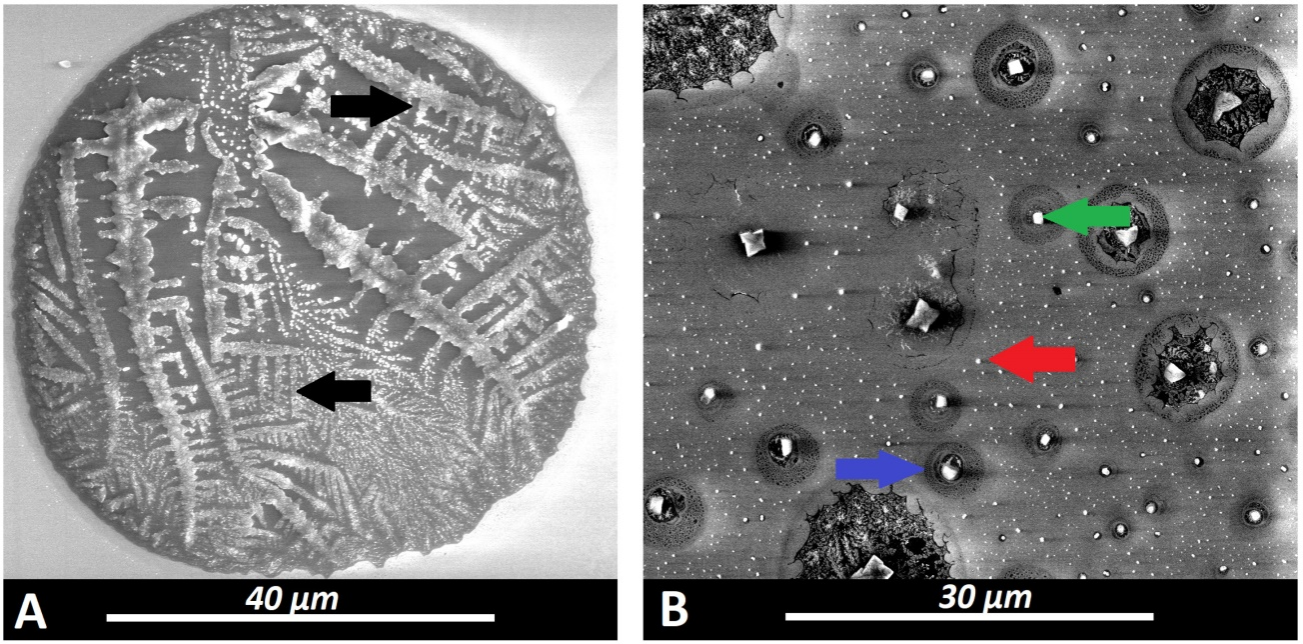
Scanning electron microscopy (SEM) of (A) remnants of a doxorubicin aerosol particle on the glass slide created by IAG via the micropump (magnification 4575) versus (B) remnants of a doxorubicin aerosol particle and nanocrystals hitting the glass slide created by EAG (magnification 5807). Black arrows (A): 2-dimensional remnants structure of drying process of “lower” concentrated chemoaerosol particles on the glass slide. Blue arrow (B): Remnant of a “superconcentrated” chemoaerosol particle with a centralized cubic crystal. Green arrow (B): central cubic crystal. Red arrow (B): Nanocrystallized chemoparticle.

**Figure 4:**
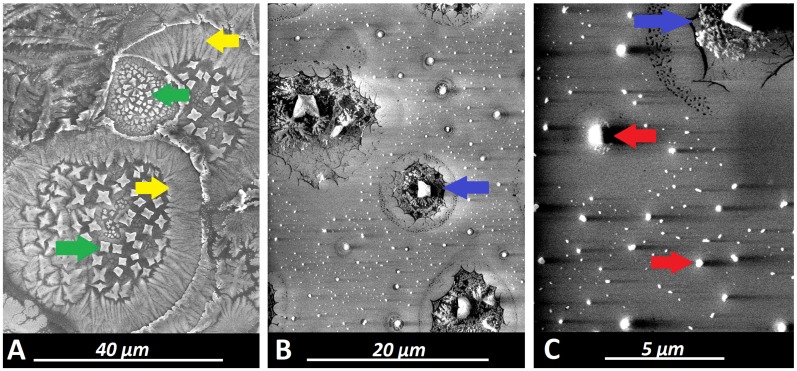
Scanning electron microscopy (SEM) of doxorubicin aerosol particles created by an EAG (Medisana 500®) using different lengths (X) of connecting tubes.(A) highly concentrated micro/nanoaerosol particles at X = 5 cm (magnification 3932). Green arrow: central cubic crystals within the remnant of a large size “superconcentrated” chemoaerosol particle. Yellow arrows: rim of the large size “superconcentrated” chemoaerosol particle. (B) X = 20 cm (magnification 7530). Blue arrow: Remnant of a “superconcentrated” chemoaerosol particle with a single centralized cubic crystal. (C) nanocrystals at X = 40 cm (magnification 19.958). Blue arrow: Remnant of a “superconcentrated” chemoaerosol particle with a single centralized cubic crystal. Red arrow: Nanocrystallized chemoparticle.

SEM measurement of aerosol particles applied by PIPAC via micropump revealed mean impact diameters of 94 +/- 55µm on the probe. These particles were mostly uniform in their appearance despite variations in their impact diameters (Figure 4A). Some had a small single crystal particle at their center which was far smaller than the diameter of the impact area itself. As visualized by SEM, the surface of the impact area demonstrates a thin layer of dried and crystallized material (Figure 2A). On the probe surface where particles were applied via EAG, particle structure differed depending on the length of the connecting tube with increased distance to the EAG. Particles larger than 30µm created a residual “fluid rim” (Figure 3A). These larger particles were mainly located at a 5 cm distance from the EAG. The fluid rim is structurally similar to the particle surface observed in PIPAC remnants dried on the probe. This appearance on surfaces is a known aspect of the crystallization of fluid solutions.

However, the center of the same particles showed rather cubic formed crystals with increasing size towards the center (Figure 3A). The amount of these larger and more complex structures was lower with increasing length of the connecting tube. Particles less than 1µm in size showed no rim formation. Particles without a rim did not hit the surface as a fluid aerosol but rather as aerosol nanocrystals, and they were also more numerous than fluid aerosol particles. An increase in connecting tube length was associated with an increase in the amount of nanocrystal particles in relationship to the aerosol droplets (Figure 3A and 3B). These were mainly observed at 20 cm and 40 cm distance to the EAG.

While particle sizes varied between 1-30 µm, all displayed visible rim formation at the impact side. However, the structure of this rim varied significantly depending on the particle size. In smaller particles the observed rims were structurally much more complex (Figure 3B and 4B) and significantly varied from the previously described, larger particles (>30 µm) and their respective rim formation. This effect is currently inexplicable.

Rim formation is considered an indicator for fluid aerosol impaction with probe surfaces (Figure 4B). Particles smaller than 1 µm do not display any rim formation and must therefore have impacted as crystallized nanoparticles. The percentual distribution of particle size in relation to total particle amount was analyzed (Figure 4B) and further specified for particles <250 nm threshold (Figure 4C).

## Conclusion

With the introduction of PIPAC less than 10 years ago, chemoaerosol therapy has experienced large-scale implementation in the clinical setting. While there are many clinical PIPAC studies, research on transport of aerosol particles remains limited. Although attempts have been made to analyze the microstructure and behavior of chemoparticles 11, 21, these efforts have been partially obstructed due to challenges in size detection technology.

However, data from many studies investigating the effect of chemoaerosol particles indicates a variety in behavior patterns and pharmacologic properties 22, 23. The introduction of nanoaerosols with superior qualities over conventional PIPAC regarding their interaction with the peritoneal surface is the latest finding in this respect 15. Currently, numerous studies are conducted to analyze evaporation effects and nanoparticle agglomeration in physical chemistry 18, 19, 24.

This study is the first to analyze chemotherapeutic particles created in INAT via SEM. These analyses offer an explanation as to why tissue penetration rates in e.g. HINAT surpass the results with classical PIPAC, which in turn displays an improvement over conventional IPC. Nanocrystallization seems to offer a new highly effective form of drug application. To our knowledge, nanocrystallization has not been described before for chemotherapeutic use. Nanocrystallized chemotherapy is a new, advanced modality for IPC specifically and chemotherapy in general. These particles demonstrate superior drug release by means of diffusion while utilizing low total drug dosages 5. The diffusion flux of a substance knowingly increases with increased concentrations according to Fick`s law of diffusion.


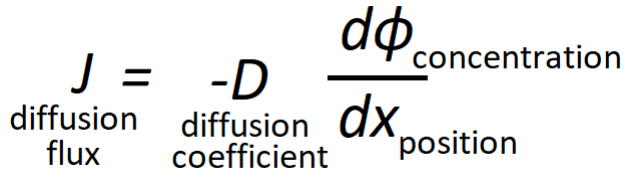


Nanocrystals can thus maximize the diffusion flux by creating a nanocrystal layer. This effect is achieved without the need to increase the total drug application dose. EAG can create nanocrystals from small drug carrying particles using evaporation effects while these are floating in the air.

**Figure 5:**
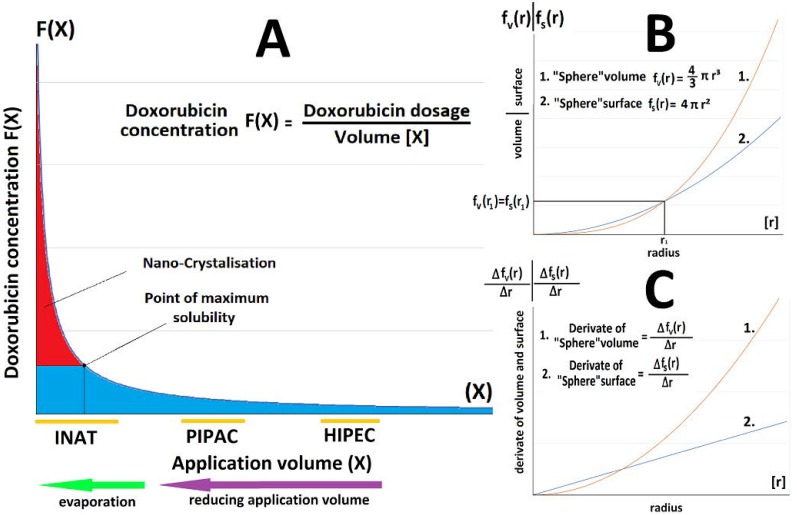
(A) Relationship between reduction in drug concentration (F(X)-axis) and increase in volume of dissolvent (X-axis) at constant dosage, demonstrated on 3 types of intraperitoneal chemotherapy. (B) Relationship between ratio of sphere volume to surface and increase in particle radius. (C) Relationship between ratio of derivatives of sphere volume to surface and increase in particle radius.

Nanocrystal formation occurs within fluid aerosol particles at a point where maximum solubility is surpassed as presented in the model (Figure 5A). This nanocrystal formation begins in smaller fluid aerosol particles as their relation between their “sphere” surface and “sphere” volume mathematically benefits evaporation effects. The relation of volume and surface regarding the particle radius (Figure 5B) as well as growth rate of volume and surface has been visualized in a graph (Figure 5C).

This manuscript presents a new form of drug particles and their possible applicational advantage in the treatment of surface malignancies. Nanocrystallized chemotherapy can present a significant advancement of current liquid or aerosol applications. However, whether this technology is limited to doxorubicin or may also be applicable using other chemotherapeutic agents remains unknown and requires further study. While this new form of nanotechnology and its potential benefits seem promising, further investigations are required to evaluate its efficacy.
